# Supplementary material for: Cryo-EM structure of an active bacterial TIR–STING filament complex
Source: Nature. Author manuscript; Available in PMC 2022 Aug 29. (PMC9402430; doi:10.1038/s41586-022-04999-1)
Supplement: SI Figure 1 [file NIHMS1825970-supplement-SI_Figure_1.pdf]

nature portfolio

https://doi.org/10.1038/s41586-022-04999-1

Supplementary information

Cryo-EM structure of an active bacterial TIR–STING filament complex

In the format provided by the
authors and unedited

Supplementary Figure 1 | Raw EMSA images.

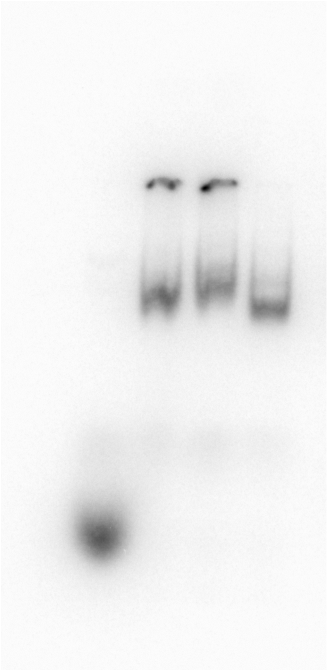

Raw (no background signal reduction/contrast correction) EMSA (Extended Data Figure 7c)

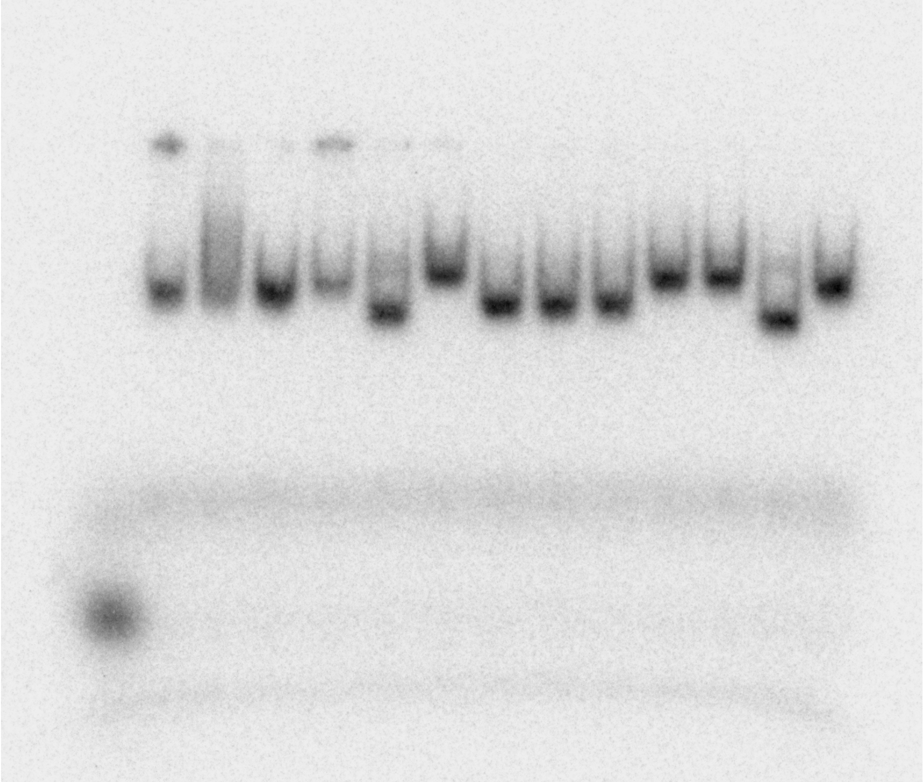

Raw (no background signal reduction/contrast correction) EMSA (Extended Data Figure 7d)
