# Supplementary material for: Cryo-EM structure of an active bacterial TIR–STING filament complex
Source: Nature. 2022 Jul 20;608(7924):803–7. doi: 10.1038/s41586-022-04999-1 (PMC9402430; doi:10.1038/s41586-022-04999-1)
Supplement: Supplementary file 1 [file 41586_2022_4999_MOESM2_ESM.pdf]

nature portfolio

Corresponding author(s): Philip J. Kranzusch & Sichen Shao

Last updated by author(s): May 24, 2022

Reporting Summary

Nature Portfolio wishes to improve the reproducibility of the work that we publish. This form provides structure for consistency and transparency in reporting. For further information on Nature Portfolio policies, see our Editorial Policies and the Editorial Policy Checklist.

Statistics

For all statistical analyses, confirm that the following items are present in the figure legend, table legend, main text, or Methods section.

n/a	Confirmed	
☐	☒	The exact sample size (n) for each experimental group/condition, given as a discrete number and unit of measurement
☒	☐	A statement on whether measurements were taken from distinct samples or whether the same sample was measured repeatedly
☐	☒	The statistical test(s) used AND whether they are one- or two-sided<br>*Only common tests should be described solely by name; describe more complex techniques in the Methods section.*
☒	☐	A description of all covariates tested
☒	☐	A description of any assumptions or corrections, such as tests of normality and adjustment for multiple comparisons
☐	☒	A full description of the statistical parameters including central tendency (e.g. means) or other basic estimates (e.g. regression coefficient) AND variation (e.g. standard deviation) or associated estimates of uncertainty (e.g. confidence intervals)
☐	☒	For null hypothesis testing, the test statistic (e.g. F, t, r) with confidence intervals, effect sizes, degrees of freedom and P value noted<br>*Give P values as exact values whenever suitable.*
☒	☐	For Bayesian analysis, information on the choice of priors and Markov chain Monte Carlo settings
☒	☐	For hierarchical and complex designs, identification of the appropriate level for tests and full reporting of outcomes
☒	☐	Estimates of effect sizes (e.g. Cohen's d, Pearson's r), indicating how they were calculated

Our web collection on statistics for biologists contains articles on many of the points above.

Software and code

Policy information about availability of computer code

Data collection	SerialEM 3.8.5 and 3.8.6
Data analysis	Phenix 1.17, Coot 0.8.9, PyMOL 2.5.1, GraphPad Prism 9.3.0, RELION 3.1, cryoSPARC 3.1.018, Chimera 1.2.5, MotionCor2 1.4.0, GCTF 1.06

For manuscripts utilizing custom algorithms or software that are central to the research but not yet described in published literature, software must be made available to editors and reviewers. We strongly encourage code deposition in a community repository (e.g. GitHub). See the Nature Portfolio guidelines for submitting code & software for further information.

Data

Policy information about availability of data

All manuscripts must include a data availability statement. This statement should provide the following information, where applicable:

- Accession codes, unique identifiers, or web links for publicly available datasets
- A description of any restrictions on data availability
- For clinical datasets or third party data, please ensure that the statement adheres to our policy

Data that support the findings of this study are available within the article and its Extended Data, Supplementary Data, and Source Data. Protein Data Bank (PDB) database accessions are listed where appropriate. All generated cryo-EM structure data have been deposited to the Protein Data Bank (PDB) database and will be made available upon public release of this manuscript. PDB accession codes are as follows: SfSTING single fibers with c-di-GMP- 7UN8 and EMD-26616, SfSTING double fibers with c-di-GMP- 7UN9 and EMD-26617, SfSTING short fibers with 3′,3′-cGAMP (masked)- 7UNA and EMD-26618, and SfSTING short fibers with 3′,3′-cGAMP- EMD-26619. PDB 6WT5 was used as a starting model for docking and building the SfSTING structure into the cryo-EM maps.

Field-specific reporting

Please select the one below that is the best fit for your research. If you are not sure, read the appropriate sections before making your selection.

☒ Life sciences ☐ Behavioural & social sciences ☐ Ecological, evolutionary & environmental sciences

For a reference copy of the document with all sections, see nature.com/documents/nr-reporting-summary-flat.pdf

Life sciences study design

All studies must disclose on these points even when the disclosure is negative.

Sample size	No sample size calculation was performed and this generally is not relevant for biochemical and bacterial growth experiments. Bacterial cultures were grown to optical density (OD600) within linear range of absorbance measuring instrumentation.
Data exclusions	No data were excluded from the analyses.
Replication	All experiments were performed with independent replicates as described and based on widely adopted protocols in the field (2-3 technical replicates per experiment, >2 independent biological replicates). All attempts at replication were successful within expected variation.
Randomization	Randomization is not relevant to the biochemical, structural, and bacterial growth experiments described in this work as this would not impact the interpretation of the results collected.
Blinding	Data were not blinded. Blinding is not relevant to the experiments described in this work as subjective analyses were not performed and blinding would not impact the interpretation of the results collected.

Reporting for specific materials, systems and methods

We require information from authors about some types of materials, experimental systems and methods used in many studies. Here, indicate whether each material, system or method listed is relevant to your study. If you are not sure if a list item applies to your research, read the appropriate section before selecting a response.

Materials & experimental systems

n/a	Involved in the study	
☒	☐	Antibodies
☒	☐	Eukaryotic cell lines
☒	☐	Palaeontology and archaeology
☒	☐	Animals and other organisms
☒	☐	Human research participants
☒	☐	Clinical data
☒	☐	Dual use research of concern

Methods

n/a	Involved in the study	
☒	☐	ChIP-seq
☒	☐	Flow cytometry
☒	☐	MRI-based neuroimaging
